# How digital support and digital competence influence digital resilience of college students in an online learning environment

**DOI:** 10.3389/fpsyt.2025.1689767

**Published:** 2025-10-29

**Authors:** Fangfang Pan, Guangxing Zhu

**Affiliations:** ^1^ Sports Institute, Huaqiao University, Xiamen, China; ^2^ Division of Student Affairs, Huaqiao University, Xiamen, China; ^3^ Institute of Education, Xiamen University, Xiamen, China

**Keywords:** online learning, digital resilience, digital support, digital competence, interpersonal interaction

## Abstract

With the deep integration of digital technology and education, online learning has become a primary learning method for college students. However, issues such as information overload, technological risks, and a lack of presence frequently arise, intensifying the information cocoon phenomenon, which exposes students to more uncertainties and challenges in learning process, making it urgent for them to possess digital resilience to cope effectively. The study conducted a questionnaire survey among 4894 college students from China to explore the impact mechanism of digital support and digital competence on digital resilience. SPSS 27.0 and Amos 27.0 were used to perform reliability, validity and discriminant validity tests, descriptive statistics, and structural equation modeling. Meanwhile, Mplus 8.0 was applied for multiple-indicator and multiple-cause analysis. The research results indicate that both digital support and digital competence significantly influence digital resilience. Meanwhile, interpersonal interaction, including social and academic interactions, act as mediators between digital support, digital competence and digital resilience. Furthermore, the impact of digital competence on interpersonal interaction and digital resilience is considerably higher than that of digital support. The results of multiple-indicator and multiple-cause indicate that after adding covariates, the action paths and directions between the variables remain unchanged, with only slight changes in coefficients. It proves that the model constructed has good robustness. The study reveals the formation mechanism of college students’ digital resilience in the online learning environment. It also provides empirical evidence and practical paths for enhancing students’ technical adaptability, risk response ability and continuous learning ability in the digital environment.

## 1 Introduction

The new generation of technological revolution has fostered a landscape where everything is a medium, continuously reshaping the ways humans interact with technology (1). Against this backdrop, the widespread integration of internet, artificial intelligence, big data, and other technologies into education is driving a shift in higher education pedagogy from traditional classrooms to online and blended learning models (2, 3). Online education and face-to-face education exhibit a certain degree of consistency in the quality of student development (4). Digital technologies not only facilitate students’ access to knowledge and academic exchanges but also reconfigure their learning ecosystems and interaction patterns (5). However, digital learning environments are far from risk-free or barrier-free. While college students benefit from the advantages of digital learning, they must also navigate adaptive challenges arising from shifts in learning modalities and technology use (6), including distracted attention, lack of presence, social isolation, cognitive overload, information saturation, technological iteration, privacy vulnerabilities etc. These issues may trigger negative emotions such as anxiety and frustration, and in extreme cases, lead to learning disengagement (7). Evidently, online learning environments demand enhanced comprehensive competencies from college students. The ability to maintain positive adaptation and recover quickly in the face of such challenges has become a critical measure of college students’ survival and development in the digital age.

In addressing the risks and uncertainties of digital environments, digital resilience(DR), as an extension of psychological resilience in digital contexts, emerges as a key factor supporting individuals’ adaptability and sustainable development. Scholarly discourse on digital resilience currently operates at two levels. First, at the organizational level, it refers to an organization’s capacity to adapt to environmental changes, mitigate risks and sustain development amid digital transformation and technological disruptions, representing a foundational characteristic of stable relationships between social organizations and digitally infused spaces. Second, at the individual level, it denotes an individual’s ability to resist risks when encountering crises or technical issues, extending psychological resilience into digital scenarios. Specifically, it encompasses the capacity to effectively cope with, recover from and learn to adapt and grow from situations such as technical failures, information clutter, learning interruptions, or social barriers (8, 9). This study focuses on individual-level digital resilience. In online learning, digital resilience not only motivates learners to sustain adaptive mindsets toward online environments and foster healthy interactions with peers, but also enables them to overcome technical obstacles, identify potential risks and achieve learning goals through strategies like emotional regulation and interpersonal support. It serves as a critical indicator of learning performance and sustained engagement, as well as a foundation for promoting mental well-being and lifelong learning competencies (10, 11). As a core attribute of individuals’ adaptation to external changes in the digital era (12), digital resilience empowers college students to address new technological risks through external interventions and internal self-regulation, accelerating their integration into online learning models.

Scholarly attention to digital resilience has grown in recent years. At the organizational level, research predominantly examines enterprises (13, 14), educational institutions (15), and public sectors (16–18), exploring strategies to enhance organizational risk resistance and adaptability during digital transformation. At the individual level, scholars have applied resilience theory to education, investigating the construction of students’ digital resilience, the role of resilience education in improving response capabilities, and the promotion of healthy development. Research on digital resilience covers a wide range of populations, including adolescents (19, 20), college students (21, 22), and corporate employees (23, 24). Especially, during COVID-19, digital resilience was widely emphasized (25, 26). However, existing research still has certain limitations. First, research content tends to center on foundational topics such as conceptual definitions (27), framework components (28), and cultivation strategies (29), with insufficient depth into its formation mechanisms and influencing factors. Second, empirical studies are relatively scarce, with a preponderance of theoretical discussions and qualitative analyses. It tends to remain in describing current situation, struggling to reveal the intrinsic connections between various factors.

In this regard, this article examines how digital support (DS), digital competence (DC), and interpersonal interaction (II) affect college students’ digital resilience based on empirical data from 4894 college students in China. It aims to reveal the mechanism of digital resilience formation in online learning environments, thereby providing practical guidance for educators to optimize online teaching strategies and enhance students’ resilience. Meanwhile, the study will offer theoretical and practical support for advancing the high-quality development of online education.

Specifically, focus on the following research questions.

First, what is the current status of digital resilience, digital support, digital competence and interpersonal interaction of college students?
Second, how do digital support and digital competence affect college students’ digital resilience?
Third, what role does interpersonal interaction play in the process of college students’ digital support and digital competence predicting their digital resilience?

## 2 Literature review and research hypotheses

### 2.1 Conservation of resources theory

The Conservation of Resources (COR) Theory, proposed by psychologist Hobfoll, centers on unraveling the mechanisms of continuous interaction between individuals and their environments (30). The theory posits that humans, as adaptive beings, possess an inherent motivation to acquire, retain, nurture, and protect resources to adapt to their surroundings and meet survival needs. In this theory, resources are defined as things that an individual perceives as helpful in achieving their goals (31–33), and are categorized into four types: material resources, conditional resources, personal characteristic resources and energy resources. A core mechanism of COR Theory is that threats or losses of resources cause stress, whereas resource gains, such as acquiring support or enhancing capabilities, strengthen psychological resilience, enabling individuals to cope with challenges.

From the perspective of COR theory, digital support constitutes a form of conditional resource. External support systems, including digital infrastructure, technical services, and management guarantees, directly determine the smoothness of online learning. Adequate digital support effectively minimizes learning disruptions and emotional distress caused by technical failures or poor access to information, thereby alleviating students’ stress and anxiety in digital environments. However, the effectiveness of digital support is not solely determined by its quantity. Rather, it is closely tied to students’ digital competence. Classified as a personal characteristic resource under COR theory, digital competence refers to students’ skills in efficiently accessing, processing, and applying information in digital settings. It encompasses not only proficiency in technical operations, but also advanced cognitive abilities, such as problem-solving and risk identification and mitigation. Students with strong digital competence can more effectively screen and integrate online learning resources, avoid negative impacts of technical failures and information overload on learning outcomes, and thus reduce resource waste and psychological burden.

In online learning environments, digital resilience serves as an effective buffer against negative emotions and mental health issues arising during the learning process. Specifically, digital resilience helps students cope with psychological stressors, including technical difficulties, information overload, and feelings of isolation in learning. Positive psychology is a constructor of personal resources, enabling students to enhance self-regulation and social-emotional buffering capabilities in digital contexts (34). When individuals encounter challenges, digital resilience enables them to adjust coping strategies, mobilize available resources, and optimize learning behaviors. More importantly, digital resilience functions as a protective factor in online education. When facing psychological risks such as anxiety, frustration, and alienation, it helps individuals maintain psychological balance and enhance self-efficacy. In addressing these stressors, digital resilience not only reduces the emergence of negative emotions but also strengthens individuals’ learning motivation and self-regulatory abilities, thereby improving learning effectiveness and mental health.

Essentially, digital resilience is the outcome of effective resource management. When digital support and digital competence generate a synergistic effect, individuals exhibit significantly enhanced ability to resist resource loss, which will strengthen psychological resilience, cope with setbacks, and safeguard mental health. Mere abundance of resources or prominence of individual abilities is insufficient to improve adaptability. The potential value of resources depends on the activation and application of individual abilities. Only through positive interaction between the two can optimal resource efficiency be achieved, thereby enhancing overall adaptability, innovation, and resilience. The research from the Hong Kong Polytechnic University indicates that students’ learning outcomes depend more on resource conditions and personal competencies than on a single teaching model. Similarly, the key to digital resilience and learning effectiveness lies not in the teaching model, but in students’ conditional resources and personal resources (4). Specifically, digital support provides the material foundation and institutional guarantees for online learning, but its maximum effectiveness relies on learners’ digital competence. The synergy not only improves individuals’ psychological resilience and behavioral adaptability when confronting challenges like technical failures and information overload but also strengthens sustained learning motivation and autonomous learning capabilities. Only with a certain level of digital competence can individuals fully utilize the technologies and resources provided by digital support, effectively address technical risks and information challenges in online learning environments, and ultimately enhance digital resilience.

### 2.2 Digital support, digital competence and digital resilience

Digital support refers to an individual’s comprehensive perception and evaluation of digital resources, technical services and supporting management systems provided within their educational environment (35). In the context of digital learning, adequate and stable digital support is regarded as a prerequisite for learners to effectively cope with technical challenges (36). Firstly, robust digital support can alleviate students’ frustration caused by platform instability, resource scarcity or technical failures, thereby enhancing their psychological sense of security and self-efficacy. Secondly, it provides individuals with material foundations and institutional safeguards, enabling them to quickly mobilize resources and seek technical or emotional assistance when encountering difficulties. Conversely, the absence of digital support tends to induce feelings of helplessness, avoidance behaviors, and a decline in adaptability and resilience. Thus, digital support not only constitutes a material condition in digital learning environments but also serves as a critical external driver shaping learners’ psychological security and confidence in coping with challenges.

Digital competence denotes an individual’s comprehensive ability to effectively, proficiently, and creatively utilize information technologies for information acquisition, processing, communication and problem-solving in digital contexts (37). While digital competence lays the knowledge and skill foundation for individuals to participate in digital society, digital resilience reflects the practical application of such competence when addressing digital risks and challenges. In online learning scenarios, digital competence is the core internal condition enabling students to resolve unexpected issues and maintain learning progress and quality. Individuals with strong digital competence tend to exhibit higher digital resilience when facing digital transformations and crises (38). Advanced digital competence not only allows individuals to rapidly adapt to technological iterations and platform updates, but also enables them to efficiently filter, integrate, and utilize information in an overloaded environment (39), thereby optimizing decision-making quality and learning efficiency. It follows that digital competence is both an operational prerequisite for digital learning and a key internal driver for the development of digital resilience.

Based on the above discussion, the present article suggests these hypotheses:

H1: Digital support affects digital resilience positively.
H2: Digital competence affects digital resilience positively.

### 2.3 Digital support, digital competence and interpersonal interaction

Interpersonal interaction is the dynamic process through which individuals communicate and influence with each other via language, actions etc. Online learning is a highly embedded social learning process, whose essence lies not only in the transmission of knowledge, but also in the continuous exchange and reconstruction of resources, experiences and emotions within learning communities (40). In this process, interpersonal interaction serves as a critical bridge connecting individuals and groups, encompassing two dimensions: social interaction(SI) and academic interaction(AI). Social interaction focuses on the establishment and maintenance of informal relationships, including greetings, emotional communication, etiquette expression, and loose collaboration, which help foster a safe, trusting, and harmonious group atmosphere. By contrast, academic interaction centers on the exchange of knowledge and intellectual ideas, such as academic discussions, research collaborations, conceptual clarification and opinion debates, with its core value lying in promoting cognitive co-construction and knowledge innovation.

Digital support provides the necessary technical and institutional foundations for students’ interpersonal interaction. On one hand, stable and functionally comprehensive online platforms can effectively reduce technical barriers in online communication, ensuring the smooth transmission of information between teachers, students, and peers, as well as the realization of diverse interaction formats. On the other hand, timely technical response mechanisms and institutionalized support measures can lower technical thresholds and usage costs, allowing learners to focus more on substantive academic and social exchanges rather than being hindered by operational or connectivity issues. Conversely, inadequate digital support may lead to delayed communication, reduced interaction frequency, or even the dissolution of learning communities. Technical obstacles such as information delays, audio-visual interruptions, and platform crashes can disrupt interaction processes, weaken the immediacy and immersion of communication, and thus undermine the cohesion and stability of learning communities.

Digital competence also directly affects the quality and depth of individuals’ engagement in online interactions. Learners with high digital competence can not only flexibly master various online collaboration and communication tools, but also select appropriate media and strategies based on different interaction needs, thereby improving communication efficiency and expression accuracy. In learning environments characterized by multitasking and parallel platform usage, individuals with strong digital competence are better able to balance social interaction and academic interaction, maintaining effective participation and value contribution amid complex information flows. In contrast, those with insufficient digital competence, despite potential willingness to participate, often encounter communication barriers and reduced engagement depth when facing interface complexity, frequent tool updates, or multi-platform collaboration requirements, which in turn affects their centrality and influence within interaction networks.

Based on the above discussion, the present article suggests these hypotheses:

H3: Digital support affects interpersonal interaction positively.
H4: Digital competence affects interpersonal interaction positively.

### 2.4 Interpersonal interaction and digital resilience

Interpersonal interaction serves not only as a channel for knowledge and information transmission, but also as a key mechanism for shaping learners’ psychological resilience and social support networks. In digital learning environments, interpersonal interaction can be categorized into two dimensions: academic interaction and social interaction. Academic interaction provides learners with diverse perspectives, problem-solving approaches, and thinking modes. It is conducive to enhancing individuals’ adaptability and problem-solving capabilities when tackling complex tasks and sudden technical issues. Through academic exchanges with others, individuals not only acquire new cognitive frameworks, but also resolve confusion, thereby boosting self-efficacy and sustaining learning engagement. Social interaction helps learners maintain emotional stability and self-efficacy under stressful circumstances through emotional resonance and psychological comfort (41). Such social support alleviates feelings of isolation and helplessness, preventing reduced learning investment caused by a lack of social connection. High-quality interpersonal interaction effectively mitigates negative impacts of platform technical failures, resource shortages, or task-related stress. When encountering setbacks, students can more easily seek help, share resources, and rebuild learning motivation and confidence. Furthermore, frequent and meaningful interaction fosters a sense of belonging and group identity, enabling learners to demonstrate greater psychological resilience and sustained learning persistence when facing external disruptions such as technological iterations or curriculum changes.

Based on the above discussion, the present article suggests these hypotheses:

H5: Interpersonal interaction affects digital resilience positively.

### 2.5 Digital support, digital competence, interpersonal interaction and digital resilience

As a core mechanism for resource transformation and activation, interpersonal interaction enhances students’ digital resilience in digital contexts. It provides students with emotional and cognitive support through social support, collaborative learning, and emotional communication. In this process, individuals can obtain emotional comfort and problem-solving advice via interactions with others, effectively reducing anxiety and frustration in online learning. To a large extent, the opportunities, resources, and support available to individuals in learning and work depend on the structure and quality of their social networks. In online learning scenarios, while digital support and digital competence are undoubtedly core factors in building digital resilience. The effectiveness may vary depending on the level of interpersonal interaction. Specifically, even if learners have access to high-quality technical conditions and strong digital competence, they may struggle to effectively mobilize potential resources when facing technical and academic pressures in environments with scarce interpersonal interaction. It can stem from a lack of emotional support, information isolation, or limited opportunities for collaboration. Conversely, in learning communities characterized by frequent and high-quality interaction, individuals can gain multiple benefits across technical, cognitive, and emotional dimensions. Technical problems can be quickly resolved through collective knowledge, cognitive dilemmas can be overcome via group wisdom, and emotional fluctuations can be buffered by social support. It not only maximizes the value of digital competence and digital support, but also directly promotes the formation and consolidation of digital resilience (25).

Based on the above discussion, the present article suggests these hypotheses:

H6: Digital support promotes the enhancement of digital resilience by mediation through interpersonal interaction.
H7: Digital competence promotes the enhancement of digital resilience by mediation through interpersonal interaction.

### 2.6 Research focus and theoretical framework

**Figure 1:**
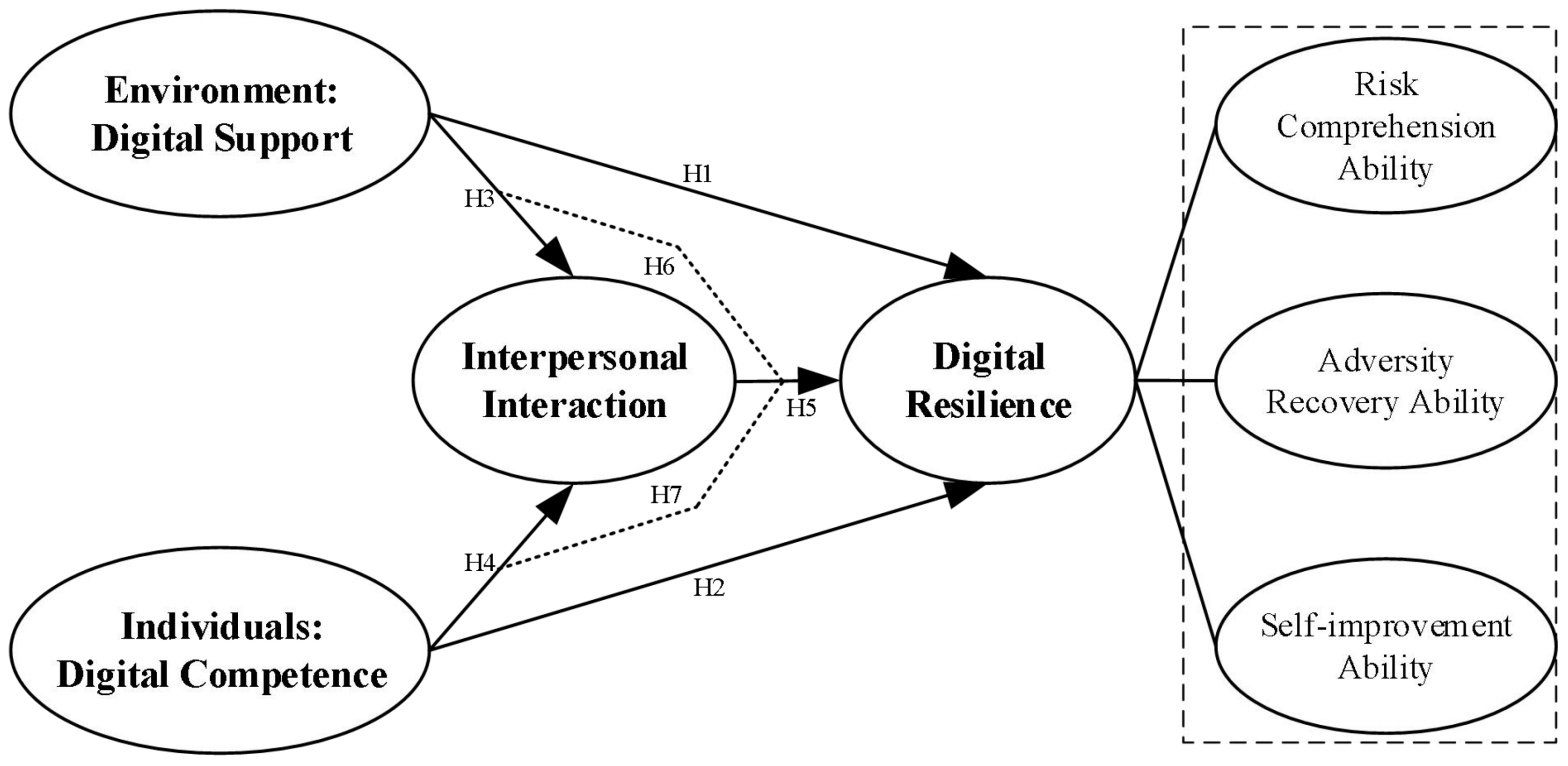
Research hypothesis diagram. digital support→digital resilience; H2: digital competence→digital resilience; H3: digital support→interpersonal interaction; H4: digital competence→interpersonal interaction; H5: interpersonal interaction→digital resilience; H6: digital support→interpersonal interaction→digital resilience; H7: digital competence→interpersonal interaction→digital resilience.

H1: digital support→digital resilience; H2: digital competence→digital resilience; H3: digital support→interpersonal interaction; H4: digital competence→interpersonal interaction; H5: interpersonal interaction→digital resilience; H6: digital support→interpersonal interaction→digital resilience; H7: digital competence→interpersonal interaction→digital resilience.

In summary, digital resilience is a key psychological and behavioral capability for students to cope with risks, adversities, and challenges in digital learning, shaped by multiple factors including digital support, digital competence and interpersonal interaction. However, previous studies have predominantly focused on enterprises, educational institutions, public sectors, adolescents, etc., with relatively limited attention to the impact of digital support and digital competence on digital resilience, and even less exploration of the mediating role of interpersonal interaction. Therefore, targeting 4894 Chinese college students, the article systematically illuminates the formation mechanism of digital resilience in online learning environments. It provides empirical evidence and practical pathways for enhancing technological adaptability, risk coping abilities, and sustained learning capacity within digital environments (see **Figure 1**).

## 3 Research methods

### 3.1 Ethics

This study employs a quantitative research approach. Ethical approval was obtained from the relevant institutional review board. All participants were involved on an informed basis. Prior to the completion andants were briefed on the purpose and significance of the study, instructed on the requirements and precautions for questionnaire completion, and assured that their personal privacy would be strictly protected. These measures were implemented to alleviate participants’ concerns and ensure the authenticity and validity of the collected data.

### 3.2 Participants

The study adopted the convenience sampling method and conducted surveys at universities in China, including Shandong University, Shenyang Pharmaceutical University, Huaqiao University, Jiangxi University of Science and Technology, Hubei University, and Ningbo University etc., which covered different regions of China. It can help reduce the interference of regional differences on the research results. Meanwhile, these universities include different types, such as comprehensive universities, science and engineering universities, and medical and pharmaceutical universities. It allows the study to take into account characteristics of college students from different university and avoid biases caused by concentrating samples on a single type of university. The participants were full-time undergraduate students, covering students of all grades and majors to improve the representativeness. The study mainly distributed questionnaires through the online platform Wenjuanxing. To ensure data validity, the study set clear exclusion criteria, including excessively short response time, and skipping or missing multiple questions. A total of 5347 questionnaires were collected. After excluding invalid responses, 4894 valid questionnaires remained, yielding an effective recovery rate of 91.53%.

Among the valid participants, 2498 (51.0%) were male and 2396 (49.0%) were female. In terms of household registration, 1982 (40.5%) held urban household registration, while 2912 (59.5%) had rural household registration. By academic year, 2496 (51.0%) were freshmen, 1536 (31.4%) were sophomores, 505 (10.3%) were juniors, 53 (1.1%) were seniors, and 304 (6.2%) were categorized as others. Regarding academic disciplines, 1726 (35.30%) were enrolled in humanities and social sciences, and 3168 (64.70%) in science and engineering.

### 3.3 Research instruments and procedures

#### 3.3.1 Research instruments

##### 3.3.1.1 Digital resilience

Digital resilience encompasses three dimensions: risk comprehension ability(RCA), adversity recovery ability(ARA), and self-improvement ability(SIA) (42, 43). This construct was measured using 13 items on a 5-point Likert scale, where responses ranged from 1 (strongly disagree) to 5 (strongly agree).

##### 3.3.1.2 Digital support

The measurement of digital support adapted from Siebert et al. (44), which form a unidimensional structure. Responses were rated on a 5-point Likert scale, where responses ranged from 1 (strongly non-consistent) to 5 (strongly consistent).

##### 3.3.1.3 Digital competence

Digital competence encompasses not only technical skills, but also cognitive, social, and emotional competencies in digital contexts (45). This variable was assessed using 5 adapted items from Ng (46) and Getenet et al. (47), structured as a single factor. A 5-point Likert scale was used, with anchors from 1 (not at all favorable) to 5 (fully favorable).

##### 3.3.1.4 Interpersonal interaction

Interpersonal interaction was measured using 8 items adapted from Kuo et al. (48) and Cho and Cho (49), consisting of two dimensions: social interaction and academic interaction. Responses were recorded on a 5-point Likert scale, ranging from 1 (not at all favorable) to 5 (fully favorable).

#### 3.3.2 Research procedures

To ensure the accuracy of the scale translation, we invited two bilingual experts with relevant field expertise to perform translation and back-translation, guaranteeing semantic consistency with the original text. Based on relevant theories and scales, we developed an initial scale and distributed it to eleven experts and scholars from universities. The experts provided feedback on the importance and appropriateness of wording for each dimension and specific item. Based on expert feedback, we revised the initial scale to form the preliminary questionnaire. To further validate the scale’s applicability, we conducted a pilot test and examined the collected data through statistical analysis. The results met all relevant standards, ultimately leading to the formal questionnaire. In terms of procedural safeguards, respondents were guaranteed anonymity and confidentiality of information. They were also informed that the questionnaire contained no standard answers. The questionnaire design dispersed the measurement dimensions of each construct to prevent respondents from identifying the underlying theoretical framework. All collected data was rigorously statistically tested to validate the questionnaire’s reliability and validity.

### 3.4 Data analysis

The study used SPSS 27.0, Amos 27.0, and Mplus 8.0 for data analysis. First, Harman’s single-factor test was conducted to examine common method bias. One common factor was specified, and confirmatory factor analysis (CFA) was performed using Amos 27.0. The poor fit of the model indicated that there was no serious common method bias. Second, Amos 27.0 was used to conduct CFA on the scales to assess the reliability and validity. Meanwhile, SPSS 27.0 was applied for Pearson correlation analysis, Heterotrait-monotrait (HTMT) ratio analysis, and descriptive statistics. Third, a structural equation model (SEM) was constructed to explore the relationships among digital support, digital competence, interpersonal interaction, and digital resilience. Finally, based on Mplus 8.0, multiple-indicator and multiple-cause analysis was carried out to test the impact of covariates on each variable, and Bootstrap method was used to test the mediating effects.

## 4 Results

### 4.1 Structural validity test

CFA was conducted on 26 items (see **Table 1**). The Kaiser-Meyer-Olkin (KMO) ranges from 0 to 1. The closer it is to 1, the stronger the correlation between variables. Results showed that all KMO values were above 0.7, ranging from 0.760 to 0.882. It indicates that each scale had excellent validity (50). Meanwhile, the factor loadings ranged from 0.860 to 0.967, which were greater than 0.5 and met the criteria for judgment. Composite reliability (CR) is a core indicator for evaluating the internal consistency of latent variables. CR ranged from 0.942 to 0.974, which were higher than 0.7. It demonstrates that the scales had good validity (51). The model fit indices were as follows: χ²/df = 15.849, p < 0.001, SRMR = 0.019, RMSEA = 0.055, GFI = 0.923, AGFI = 0.902, PNFI = 0.836, PCFI = 0.837, CFI = 0.979, and TLI = 0.975, NFI = 0.977. Overall, the χ²/df value may be relatively high due to the large-sample effect. However, indices such as SRMR, RMSEA, CFI, and TLI all met the standards (52). Therefore, the model exhibited a good fit.

**Table 1:** Reliability and validity test.

Variable	Item	Std. estimates	Std. error	P	Cronbach’s α	KMO	AVE	CR
*DS*	*DS1*	0.860	—	—	0.946	0.859	0.820	0.948
	*DS2*	0.916	0.012	P<0.001				
	*DS3*	0.948	0.014	P<0.001				
	*DS4*	0.896	0.008	P<0.001				
*DC*	*DC1*	0.904	—	—	0.963	0.878	0.869	0.964
	*DC2*	0.934	0.008	P<0.001				
	*DC3*	0.951	0.008	P<0.001				
	*DC4*	0.939	0.008	P<0.001				
*II*	*SI1*	0.903	—	—	0.941	0.760	0.845	0.942
	*SI2*	0.911	0.009	P<0.001				
	*SI3*	0.943	0.009	P<0.001				
	*AI1*	0.899	—	—	0.944	0.792	0.858	0.948
	*AI2*	0.943	0.008	P<0.001				
	*AI3*	0.936	0.009	P<0.001				
*DR*	*RCA1*	0.929	—	—	0.974	0.879	0.903	0.974
	*RCA2*	0.959	0.007	P<0.001				
	*RCA3*	0.967	0.007	P<0.001				
	*RCA4*	0.946	0.008	P<0.001				
	*ARA1*	0.882	—	—	0.956	0.871	0.846	0.956
	*ARA2*	0.931	0.011	P<0.001				
	*ARA3*	0.928	0.011	P<0.001				
	*ARA4*	0.937	0.010	P<0.001				
	*SIA1*	0.922	—	—	0.970	0.882	0.892	0.971
	*SIA2*	0.953	0.008	P<0.001				
	*SIA3*	0.950	0.008	P<0.001				
	*SIA4*	0.953	0.008	P<0.001				

### 4.2 Reliability test

Reliability refers to the consistency, stability, and dependability. Cronbach’s α coefficient is a commonly used indicator for assessing reliability. The Cronbach’s α coefficients exceeded 0.7, with specific values of 0.946, 0.963, 0.941, 0.944, 0.974, 0.956 and 0.970 (see **Table 1**). It indicates that the internal consistency of each scale is extremely high. The measurement results are stable and reliable, and the reliability is excellent (50).

### 4.3 Discrimination validity test and descriptive statistics

The study first employed Pearson correlation to test the relationships among variables. As shown in **Table 2**, all variables exhibit positive correlations in pairwise comparisons. In **Table 1**, the average variance extracted (AVE) ranged from 0.820 to 0.903, exceeding 0.5. The square roots of AVE surpassed Pearson correlation coefficients.

**Table 2:** Pearson correlation, HTMT analysis, and descriptive statistics (n=4894).

Variable	DS	DC	II-SI	II-AI	DR-RCA	DR-ARA	DR-SIA
Pearson correlation							
*DS*	**0.906**						
*DC*	0.736^**^	**0.932**					
*II-SI*	0.710^**^	0.774^**^	**0.919**				
*II-AI*	0.718^**^	0.781^**^	0.898^**^	**0.926**			
*DR-RCA*	0.674^**^	0.705^**^	0.819^**^	0.846^**^	**0.950**		
*DR-ARA*	0.703^**^	0.776^**^	0.860^**^	0.888^**^	0.864^**^	**0.920**	
*DR-SIA*	0.731^**^	0.863^**^	0.830^**^	0.843^**^	0.763^**^	0.835^**^	**0.944**
HTMT							
*DS*	—						
*DC*	0.770	—					
*II-SI*	0.753	0.813	—				
*II-AI*	0.758	0.818	0.852	—			
*DS-RCA*	0.703	0.730	0.857	0.882	—		
*DS-ARA*	0.739	0.809	0.907	0.933	0.896	—	
*DS-SIA*	0.762	0.892	0.868	0.878	0.786	0.867	—
Descriptive statistics							
*M*	4.312	4.176	4.350	4.331	4.431	4.321	4.220
*SD*	0.893	0.952	0.856	0.856	0.806	0.842	0.919
*Skewness*	-1.331	-1.017	-1.342	-1.276	-1.493	-1.193	-1.094
*Kurtosis*	1.476	0.430	1.603	1.378	2.156	1.149	0.790

*P<0.05; **P<0.01; ***P<0.001. The same notation applies hereinafter. Boldface numbers represent the square roots of AVE.

The study further used HTMT. Discriminant validity was evaluated by comparing the average correlation of indicators across different constructs with the average correlation of indicators within the same construct. As shown in **Table 2**, HTMT values for all pairs of constructs were basically less than 0.9, which indicates that the factors had good discriminant validity (53).

The means and standard deviations of each variable are presented in **Table 2**. The mean for digital support was 4.312, indicating that college students held a relatively positive comprehensive perception and evaluation of digital resources, technical services, and related aspects. The mean for digital competence was 4.176, which shows that college students had a relatively high level of ability in applying digital technologies, processing digital information, and similar tasks. The mean for social interaction was 4.350, suggesting that college students interacted with others relatively actively and maintained good social status and interpersonal relationships. The mean for academic interaction was 4.331, demonstrating that college students engaged in frequent and in-depth interactions in the academic field, showing a positive level of participation. The mean for risk comprehension ability was 4.431, indicating that college students had a relatively high level of awareness and understanding of various types of risks, and were able to identify and judge potential risks effectively. The mean for adversity recovery ability was 4.321, which means college students had strong resilience and adaptability when facing difficulties, setbacks, and other adverse situations. The mean for self-improvement ability was 4.220, showing that college students had a strong awareness of self-development and motivation for improvement, and could proactively seek opportunities to enhance their knowledge, skills, and comprehensive quality.

### 4.4 Structural equation model analysis

**Figure 2:**
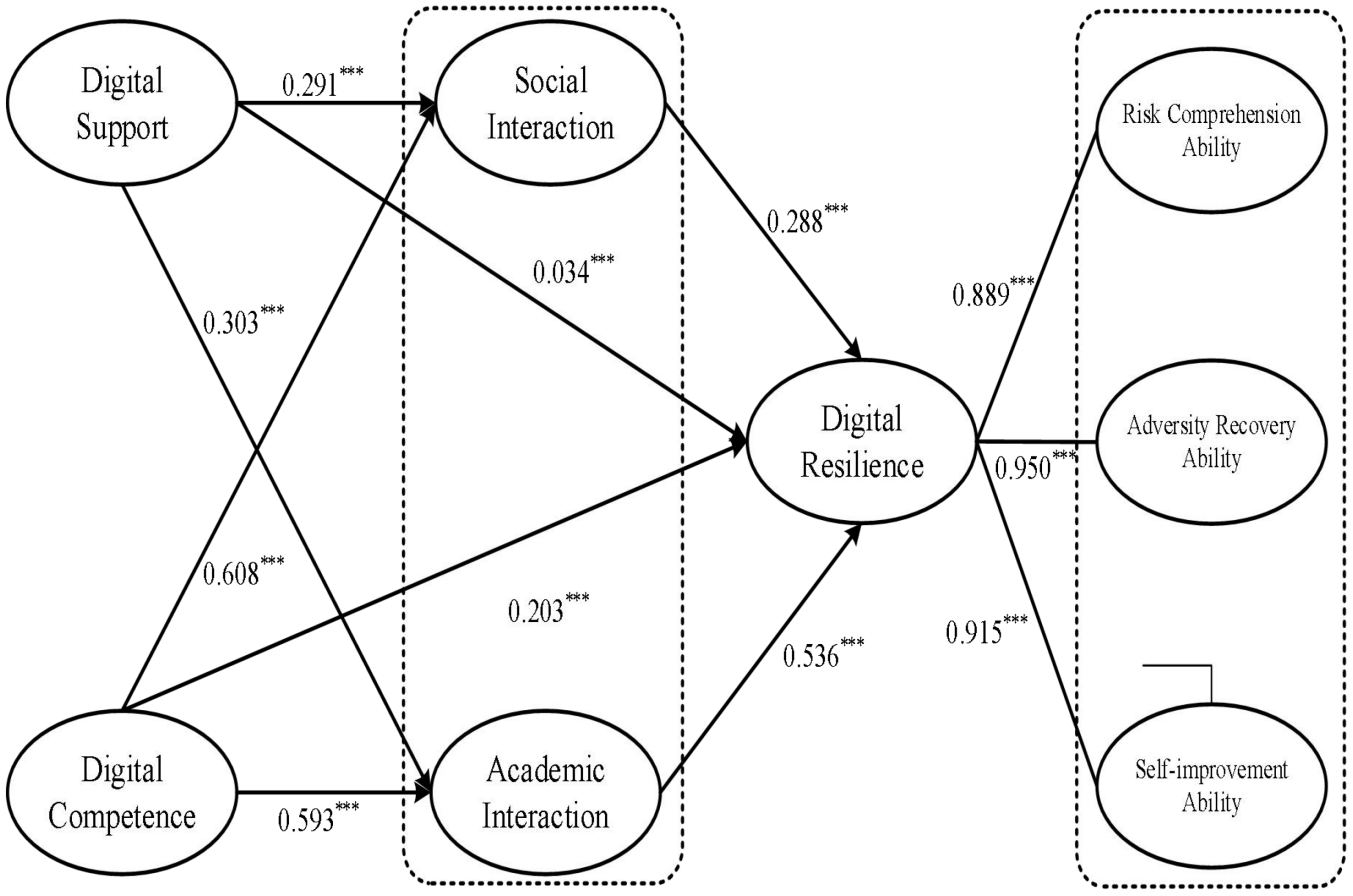
Structural equation path diagram. All coefficient values are standardized.

The study utilized Amos 27.0 to verify the influencing mechanism of college students’ digital resilience. The estimation results of the model operation converged to the proposed hypothetical model. The absolute values of skewness and kurtosis in the model were less than 3 and 10, respectively, approximating a normal distribution. **Figure 2** presents the fitting results of the structural model. All hypothetical paths were statistically significant (p < 0.001), meaning all hypotheses were supported. The SEM model showed good model fit indices, as follows: χ2/df=30.871, P<0.001, SRMR = 0.047, RMSEA = 0.078 [0.077, 0.080], GFI = 0.862, AGFI = 0.831, PNFI = 0.843, PCFI = 0.844, CFI = 0.956, TLI = 0.950, NFI = 0.954.

Digital support exerts remarkable positive effects on interpersonal interaction (social interaction: *β* = 0.291, P<0.001; academic interaction: *β* = 0.303, P<0.001) and digital resilience (*β* = 0.034, P<0.001). Hypotheses H1 and H3 are validated. Digital competence was found to provide meaningful positive effects on interpersonal interaction, namely social interaction (*β* = 0.608, P<0.001), academic interaction (*β* = 0.593, P<0.001), and digital resilience (*β* = 0.203, P<0.001). Hypotheses H2 and H4 were validated. Interpersonal interaction (social interaction: *β* = 0.288, P<0.001; academic interaction: *β* = 0.536, P<0.001) exert significantly a favorable influence on digital resilience. Hypothesis H5 is validated.

A comparative analysis of the comprehensive path coefficients reveals that digital competence generally exhibits greater influence than digital support across all paths. It suggests that in the context of online learning, individuals’ intrinsic capability factors have a more substantial impact on interaction and resilience than external environmental support factors. In other words, the degree of alignment between environmental provisions and individual capabilities constitutes the fundamental determinant of interaction quality and resilience levels in online learning environments. Regarding interpersonal interaction, digital competence exerts a significantly stronger influence on both social interaction and academic interaction compared to digital support. It indicates that while digital support provides the necessary infrastructure and technical guarantees for college students’ online learning and interactions, the key variable that truly determines learners’ active participation and the depth of interaction lies in their own digital competence. Individuals with high-level digital competence can flexibly and proficiently utilize diverse communication tools and platforms, thereby engaging in social and academic exchanges with higher frequency and quality, and forming more cohesive and efficient interaction networks. In terms of digital resilience, the direct effect of digital competence is also significantly stronger than that of digital support. It implies that although digital support can directly promote the enhancement of digital resilience to a certain extent, its mechanism of action is more likely to be reflected in indirectly facilitating the development of resilience by optimizing interaction conditions. In contrast, as an intrinsic individual trait, digital competence can be directly translated into specific strategies and actions for coping with challenges, restoring learning motivation, and adapting to changes, thereby exerting a more direct and powerful driving effect on resilience.

### 4.4 Multiple-indicator and multiple-cause model analysis

**Figure 3:**
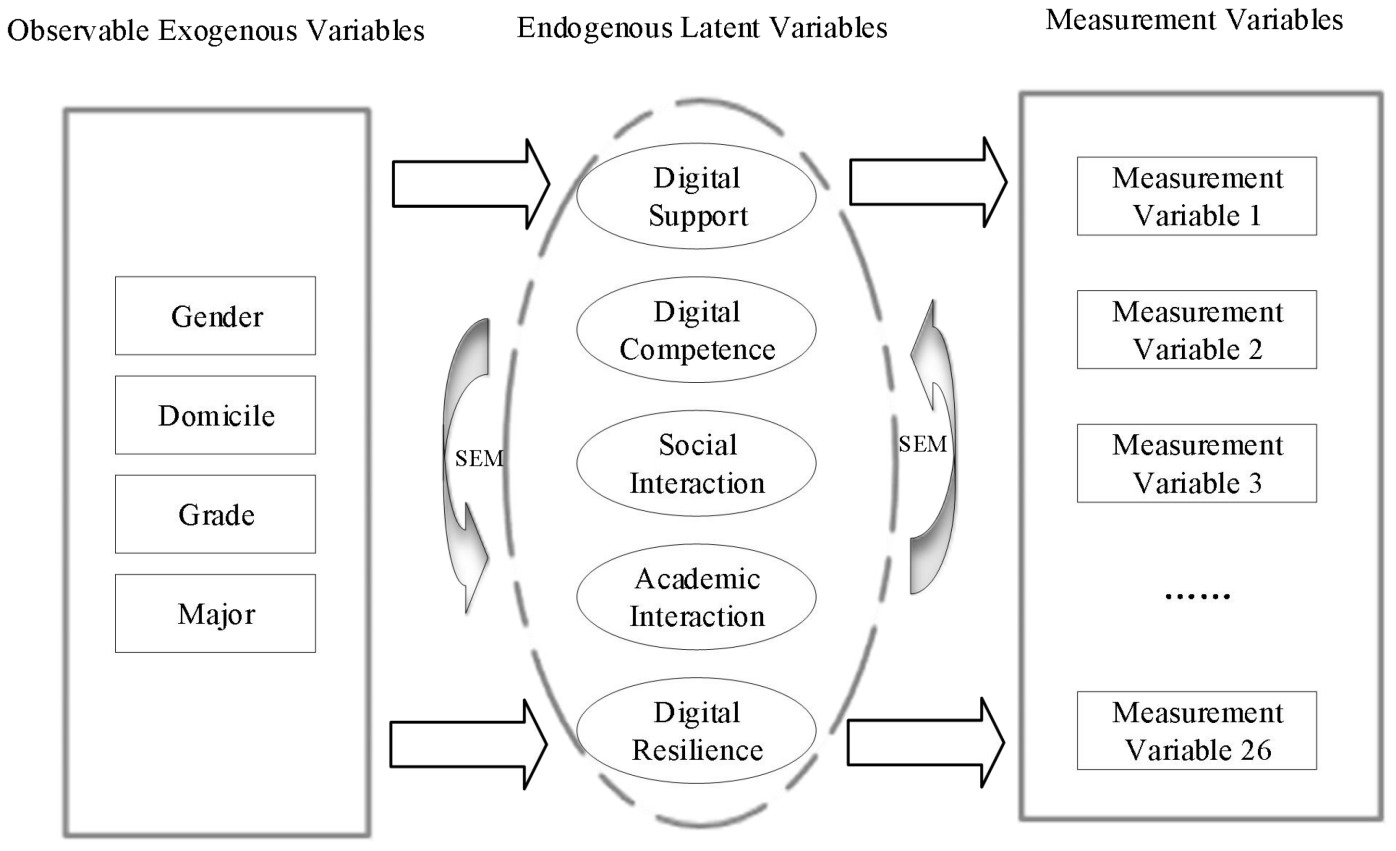
MIMIC model framework diagram.

**Figure 4:**
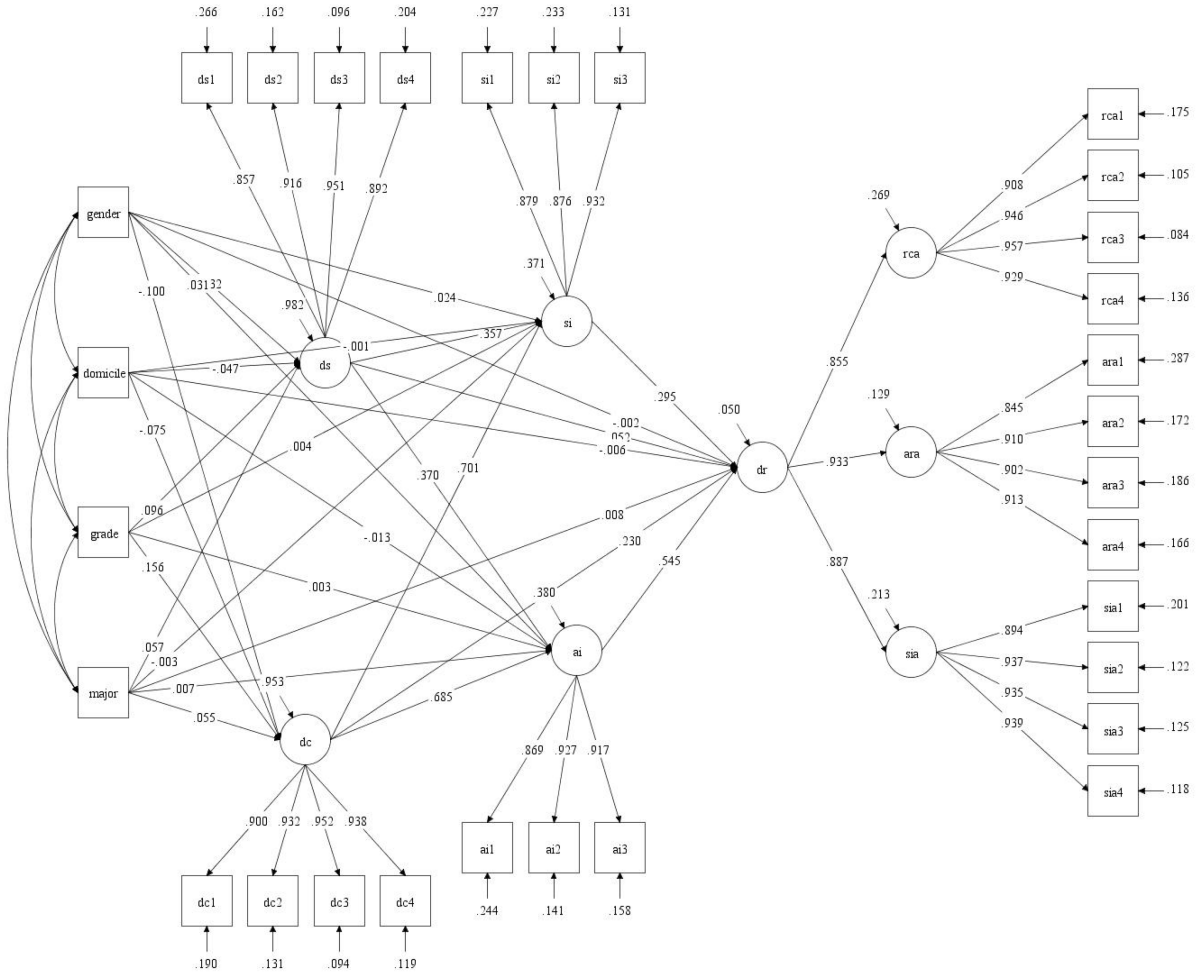
MIMIC model path analysis diagram. All coefficient values are standardized.

The study employed MIMIC model to analyze the impact of each covariate on various influencing factors of college students’ digital resilience, and also to test the robustness of the relevant model. The MIMIC model included 4 observable exogenous manifest variables, 5 endogenous latent variables, and 26 measurement variables (see **Figure 3**). On this basis, the study used Mplus 8.0, with robust maximum likelihood estimation (MLR) as the estimation method, to solve the structural relationships among observable exogenous variables, endogenous latent variables, and measurement variables. The results are presented in **Table 3**. The fitted MIMIC model demonstrated favorable fit indices: χ² = 4629.610, df = 372, RMSEA [90% CI] = 0.048 [0.047, 0.050], CFI = 0.935, TLI = 0.925. As shown in **Figure 4** and **Table 3**, after incorporating covariates, the effect paths and directions among variables in the MIMIC model of college students’ digital resilience remained unchanged, with only slight variations in effects. It indicates that the model constructed in this study has good robustness.

**Table 3:** MIMIC model of digital resilience of college students.

Path	*Std. β*	*S.E.*	*P*
*DS→DR*	0.052	0.017	<0.001
*DS→SI*	0.357	0.032	<0.001
*DS→AI*	0.370	0.032	<0.001
*DC→DR*	0.230	0.029	<0.001
*DC→SI*	0.701	0.024	<0.001
*DC→AI*	0.685	0.025	<0.001
*SI→DR*	0.295	0.031	<0.001
*AI→DR*	0.545	0.033	<0.001

As can be seen from **Table 4**, covariates do not significantly influence every latent variable in the MIMIC model. Gender (*β* = -0.032, p < 0.05), domicile (*β* = -0.047, p < 0.001), grade (*β* = 0.096, p < 0.001), and major (*β* = 0.057, p < 0.001) were influencing factors of digital support. Gender (*β* = -0.100, p < 0.001), domicile (*β* = -0.075, p < 0.001), grade (*β* = 0.156, p < 0.001), and major (*β* = 0.055, p < 0.001) were influencing factors of digital competence. Gender was an influencing factor of interpersonal interaction, namely social interaction (*β* = 0.024, p < 0.05) and academic interaction (*β* = 0.031, p < 0.05).

**Table 4:** Results of covariates on the MIMIC model of digital resilience of college students.

Variable	Gender	Domicile	Grade	Major
*DS*	-0.032^*^ (0.016)	-0.047^***^ (0.015)	0.096^***^ (0.014)	0.057^***^ (0.016)
*DC*	-0.100^***^ (0.016)	-0.075^***^ (0.014)	0.156^***^ (0.013)	0.055^***^ (0.016)
*SI*	0.024^*^ (0.011)	-0.001 (0.010)	0.004 (0.009)	-0.003 (0.011)
*AI*	0.031^**^ (0.012)	-0.013 (0.010)	0.003 (0.009)	0.007 (0.012)
*DR*	-0.002 (0.007)	-0.006 (0.006)	0.001 (0.006)	0.008 (0.007)

On the basis of controlling for covariates using the MIMIC model, the study tested the mediating effect of interpersonal interaction by performing 5000 repeated samplings with the bias-corrected percentile Bootstrap method (95% confidence interval). The results are presented in **Table 5**. Notably, social interaction and academic interaction functioned as parallel mediating variables, both falling under the category of interpersonal interaction. Social interaction and academic interaction respectively mediated the path from digital support to digital resilience, with effect values of 0.105^***^ (95% CI: 0.084, 0.132) and 0.202^***^ (95% CI: 0.168, 0.240). In other words, interpersonal interactions partially mediate the path from digital support to digital resilience. Hypothesis H6 is supported. Similarly, social interaction and academic interaction respectively function as mediators in the path where digital competence affects digital resilience, with effect values of 0.207^***^ (95% CI: 0.172, 0.245) and 0.373^***^ (95% CI: 0.333, 0.416). In other words, interpersonal interaction partially mediates the path from digital competence to digital resilience. Hypothesis H7 is validated. It can be concluded that digital support and digital competence can directly influence digital resilience, and can also impact digital resilience indirectly through mediation by interpersonal interaction, namely, social interaction and academic interaction, respectively.

**Table 5:** Mediation effect analysis in the MIMIC model.

Path	Std. β	S.E.	P	Bootstrap 5000 times 95% CI		Effect ratio
				Lower	Upper	
*DS→SI→DR*	0.105	0.015	<0.001	0.084	0.132	29.25%
*DS→AI→DR*	0.202	0.022	<0.001	0.168	0.240	56.27%
*Total Indirect Effect*	0.307	0.029	<0.001	0.261	0.357	85.52%
*Direct Effect*	0.052	0.017	<0.001	0.025	0.082	14.48%
*Total Effect*	0.359	0.029	<0.001	0.311	0.407	100%
*DC→SI→DR*	0.207	0.022	<0.001	0.172	0.245	25.56%
*DC→AI→DR*	0.373	0.025	<0.001	0.333	0.416	46.04%
*Total Indirect Effect*	0.580	0.027	<0.001	0.537	0.626	71.60%
*Direct Effect*	0.230	0.029	<0.001	0.183	0.278	28.40%
*Total Effect*	0.810	0.020	<0.001	0.777	0.841	100%

## 5 Research conclusions and discussion

Empirical data reveals that college students exhibit high levels of digital resilience, digital support, digital competence, and interpersonal interaction, with all mean scores exceeding 4. It suggests that in the digital era, they possess a solid digital foundation. On one hand, they have access to adequate digital support resources and strong digital operation skills. On the other hand, their robust digital resilience enables them to effectively identify risks in the digital environment and cope with related adversities. Additionally, digitalization provides a medium for interactions. By leveraging digital media, students can integrate into digital life and engage in learning and social activities. Using SPSS 27.0, AMOS 27.0, and Mplus 8.0 software, the study conducted an empirical analysis on 4894 college students in China. The results showed that both digital support and digital competence significantly influence digital resilience. And interpersonal interaction, including social and academic interactions, act as mediators between digital support, digital competence and digital resilience.

First, digital support and digital competence markedly affect digital resilience positively. The former emphasizes the critical role of external support in buffering stress and enhancing coping effectiveness, while the latter highlights that resilience development depends not only on individuals’ internal resources, but also on external situational resources. It aligns with previous research findings. Castillo de Mesa and Gómez Jacinto (54) surveyed young graduates in Spain and found a remarkable correlation between digital competence and digital resilience. Eri et al. (25) empirically demonstrated that digital support is crucial for students to maintain strong adaptability when engaging with diverse digital learning platforms. Cassaretto et al. (55) demonstrated that disadvantages caused by insufficient technical resources and infrastructure communication systems lead to student frustration, hinder full participation in academic activities, and ultimately affect mental health. In online learning environments, digital support and digital competence can effectively alleviate students’ perception of resource loss in study and life, and reduce the stress responses caused by such loss. The former minimizes cognitive load by reducing friction in technology use (56), while the latter enhances students’ confidence and efficiency in navigating digital environments autonomously, ultimately lowering anxiety and burnout (57). It is evident that digital transformation of higher education should not be limited to the accumulation of technical hardware. Instead, it should focus more on the systematic cultivation of students’ digital competence, enabling them to grow into digital citizens with strong adaptability and creativity. Concrete measures can be taken in two aspects. Conduct targeted digital competence training focused on information filtering, platform fluency, and risk identification to improve students’ relevant abilities. Provide timely, stable, and low-friction digital support services to reduce ineffective energy consumption of students in technology use. A hybrid model that combines reliable low-friction platforms with a design for interactive scaffolding. it allows students to fully leverage the strengths of both online and face-to-face instruction, ensuring the efficiency of technical support and the sustainability of learning interactions, thereby further optimizing the student learning experience (4).

Second, digital support and digital competence affect interpersonal interaction remarkably. The frequency and quality of interaction directly affect information flow and resource acquisition. External support lowers the threshold for participation, while internal competence improves the interaction experience. Relevant studies support this conclusion. Alshammary and Alhalafawy (58) found that college students’ perceptions of digital platforms make a significant difference in education. Zhu et al. (59) empirically confirmed that in online learning environments, the more sufficient digital support students perceive, the higher the likelihood they will engage in interpersonal interaction. Meanwhile, students with higher digital competence find it easier to operate digital tools and conduct interpersonal interaction. Digital support optimizes the usability and stability of platforms, providing a technologically accessible interactive environment (60) that can reduce the technical and psychological thresholds for students to participate in interactions. For example, rich learning platforms and real-time technical assistance can reduce delays, freezes, and information interruptions in communication, thereby increasing students’ willingness to participate (61). Pan et al. (62) also found that peer interaction in the digital environment is positively correlated with students’ academic performance and digital skills. At the same time, digital competence enhances students’ ability to master technology and operate tools proficiently, allowing them to handle complex platform functions with ease. In summary, digital support and digital competence significantly improve the quality and depth of interaction. External support reduces the cost of initiating interactions, while internal competence ensures the smoothness and effectiveness of the interaction process. It helps build trust, strengthen cooperative tendencies, and accumulate social capital. Universities should construct online teaching platforms with high stability, strong compatibility, and high interactivity. They should provide students with operational support across multiple platforms and tools, create a convenient and smooth online communication environment, and enhance students’ willingness and efficiency in participating in interactions.

Third, interpersonal interaction remarkably affect digital resilience. The findings further indicate that enhanced levels of interpersonal interaction significantly strengthen college students’ digital resilience. The higher the level of interpersonal interaction is, the stronger the digital resilience of college students becomes. It is consistent with the findings of Zhong and Ning (63) and Ding et al. (64), who both argued that active interpersonal interaction in the digital environment helps alleviate individuals’ psychological anxiety and promote mental health. Interactive networks not only provide individuals with instantly sharing of information, strategies, and skills, but also reduce psychological stress through emotional support. De et al. (65) noted that active social interaction via digital devices helps reduce psychological problems among adolescents. In digital contexts, interpersonal interaction provides students with critical socio-emotional buffering and cognitive co-regulation mechanisms that support resilience and sustained engagement. Academic interaction can quickly fill information gaps and technical shortcomings, guiding students to maintain participation. Emotional connections and a sense of belonging in social interaction help reduce feelings of isolation and frustration (66) and alleviate students’ psychological stress. High-quality interpersonal interaction is not only a channel for transmitting information and skills, but also an important social mechanism for the formation and enhancement of digital resilience. Even if individuals have certain resources and abilities, those lacking effective interaction may struggle to convert their potential into stable coping capabilities due to insufficient timely feedback and emotional support. Therefore, in curriculum design, teachers can appropriately incorporate high-quality collaboration and discussion sessions and construct designed interactional scaffolds that balance social and academic interaction with clear roles and teacher moderation. They should also encourage students to establish stable communication relationships, so that students can obtain continuous support in terms of information, emotions, and strategies.

Fourth, interpersonal interaction significantly mediates the relationship between digital support, digital competence, and digital resilience. It proves consistency with equivalence theory. similar to face-to-face and blended learning models, online learning is committed to providing an equivalent learning experience to achieve equivalent outcomes in educational expectations. When digital support and digital competence are sufficient, students can maintain interpersonal interactions and digital resilience (4). The formation of knowledge and abilities depends on social interaction contexts. In online learning environments, interpersonal interaction serves as a key behavioral pathway for converting resources and abilities into resilience. The study found that digital support provides students with platform guarantees, and digital competence equips students with the operational ability to effectively use these platforms. However, these resources and abilities do not automatically transform into resilience. They must be activated and amplified through interpersonal interaction. In contexts lacking interaction, even with sufficient resources and abilities, the process of resilience formation may be weakened. Meanwhile, cross-cultural communication is an important form of interpersonal interaction. Especially in the era of globalization, cross-cultural communication is needed to collectively strengthen digital resilience. The synergistic effect of digital support, digital competence, and interpersonal interaction enables college students to maintain resilience when facing insufficient resources or increased pressure, and sustain their participation through active contextual adaptation. Although the study found that the correlation between digital competence and digital resilience is stronger than that between digital support and digital resilience, digital support remains crucial. Digital support is more likely to function by creating favorable interaction conditions and usage environments. The design of platforms and services plays a fundamental role in maintaining resilience and promoting participation. Universities should establish systematic digital literacy training mechanisms while continuously optimizing digital learning environments. They should promote brief, culturally adaptive positive psychology practices such as short mindfulness breaks, structured gratitude reflections linked to online collaboration, and routine daily habits that foster positive emotions and social presence (34). Meanwhile, actively guide students in cross-cultural communication to enhance their digital mental health through interaction. It not only improves the quality of social and academic engagement but also builds students’ digital resilience, thereby reducing anxiety and feelings of isolation.

## 6 Research contribution and limitation

The empirical results indicate that college students have relatively sufficient digital support and a high level of digital competence. In online learning environments, college students can use digital resources appropriately, actively engage in interpersonal interaction based on their existing digital competence. They possess strong abilities in risk comprehension, adversity recovery, and self-improvement. Statistical analysis further shows that both digital support and digital competence affect digital resilience directly, and they can also influence digital resilience indirectly through interpersonal interaction. Among these factors, the influence of digital competence is generally stronger than that of digital support across all paths. It suggests that universities should not only focus on providing digital resources, but also attach greater importance to the systematic cultivation of students’ digital competence. The conclusions not only enrich the theoretical explanation of formation mechanism of digital resilience, but also provide empirical evidence and strategic guidance for digital transformation of higher education. In the future, universities should make coordinated efforts in both system design and competence cultivation. By optimizing the digital support system, strengthening the cultivation of digital competence, and promoting high-quality interpersonal interaction, they can foster digital citizens with innovative spirit and adaptability.

However, the study also has certain limitations. First, there may be inadequacies in data collection, which could lead to residual bias. And the data are mainly based on students’ self-reports. Although statistical tests have been conducted, it is difficult to avoid recall bias and reporting bias that may arise from ceiling effects or social desirability bias. In the future, questionnaire design can be optimized by broadening response ranges, including more challenging items, and balancing keyed items. A multi-source data collection strategy can be adopted, combining students’ self-assessment data with evaluations from teachers and peers. Second, the study uses cross-sectional data, which have relatively low evidential strength in causal inference. Future studies may consider longitudinal tracking or experimental interventions. Third, the study mainly uses a convenience sampling method. While this method saves time and effort and allows for quick data collection, its objectivity may be limited. Future research could incorporate stratified sampling or qualitative methods. Fourth, while this article examined the effects of digital support, digital competence, and interpersonal interaction on digital resilience, other potential factors may exist, such as baseline anxiety, perceived stress, and prior online learning experience. Future research should delve into these areas. Fifth, incorporating student mental health outcomes into digital resilience assessment framework could enhance the objectivity and accuracy of research conclusions. Finally, the study sample originated from college students in China. The generalizability and applicability of the model in other countries require further validation.

## Data Availability

The raw data supporting the conclusions of this article will be made available by the authors, without undue reservation.

## Appendix A. Construct measurement (Example)

**Table:** Table A. Variable structure framework and connotative description (example).

Variable	Rate Format	Item Description
*Digital Support*	5-point Likert scale (1 = Strongly non-consistent, 5 = Strongly consistent)	The school provides a dedicated online learning management and service system.
		The school has assigned dedicated officers to assist with your online learning.
*Digital Competence*	5-point Likert scale (1 = not at all favorable, 5 = fully favorable)	I possess a good level of proficiency in the use of information and communication technology.
		I am proficient in using various digital devices and software for online learning.
*Digital Resilience*	5-point Likert scale (1 = strongly disagree, 5 = strongly agree)	I can identify potential cybersecurity risks that may arise in online learning.
		I can overcome the challenges of online learning.
		I can make plans to complete assignments while learning online.
*Interpersonal Interaction*	5-point Likert scale (1 = not at all favorable, 5 = fully favorable)	I can apply different social skills based on the social situation during online learning.
		I actively participate in group discussions during online learning.
